# Effects of lifestyle and glucagon-like Peptide-1 receptor agonist-based therapies on waist circumference: A systematic review and meta-analysis

**DOI:** 10.1016/j.obpill.2026.100281

**Published:** 2026-05-30

**Authors:** S. Ghosal, A. Ghosal

**Affiliations:** a Nightingale Hospital, Kolkata, India; b Aston University, Birmingham, UK

**Keywords:** Waist circumference, VAT, Body weight, BMI, Age, Meta-analysis

## Abstract

**Background and aims:**

Central obesity, commonly assessed using waist circumference (WC), is strongly associated with cardiometabolic risk. While lifestyle interventions and glucagon-like peptide-1 receptor agonist-based therapies (GLP-1RA-based therapies) reduce body weight, their effects on WC and factors influencing response remain incompletely characterised. This systematic review and meta-analysis evaluated the effects of these interventions on WC in randomised controlled trials (RCTs).

**Methods:**

Following PRISMA 2020 guidelines, we searched PubMed for RCTs published between 2010 and 2025 that reported outcomes related to WC or visceral adipose tissue in adults. Eligible interventions included diet, exercise, diet plus exercise, and GLP-1RA-based therapies. Data from 27 intervention-comparator study arms (21 RCTs; n = 3196) were pooled using random-effects models to estimate between-group mean differences in WC change. Meta-regression explored associations between baseline characteristics and WC response. Publication bias was assessed using funnel plots and Egger's test.

**Results:**

Interventions were associated with significant reductions in WC (MD: −4.36 cm; 95% CI: −4.97 to −3.74; prediction interval: −6.98 to −1.73). Subgroup analyses demonstrated significant reductions for GLP-1RATs (MD -5.93 cm; 95% CI -7.88 to −3.98) and diet-alone interventions (MD -3.46 cm; 95% CI -5.91 to −1.00). Exercise-only and diet-plus-exercise interventions showed more variable effects, and subgroup differences were not statistically significant. Exploratory meta-regression suggested potential associations between baseline anthropometric measures, age, and WC reduction, although these findings should be interpreted cautiously. WC change was positively associated with visceral adipose tissue change at the study-arm level. Funnel plots suggested possible small-study effects (Egger's p = 0.0025).

**Conclusions:**

GLP-1RA-based therapies and dietary interventions were associated with reductions in WC in adults. WC may serve as a practical anthropometric marker of intervention response, although observed associations with visceral adipose tissue were derived from study-arm-level analyses. Further studies are needed to better define predictors of treatment response.

## Graphical abstract

**Figure:**
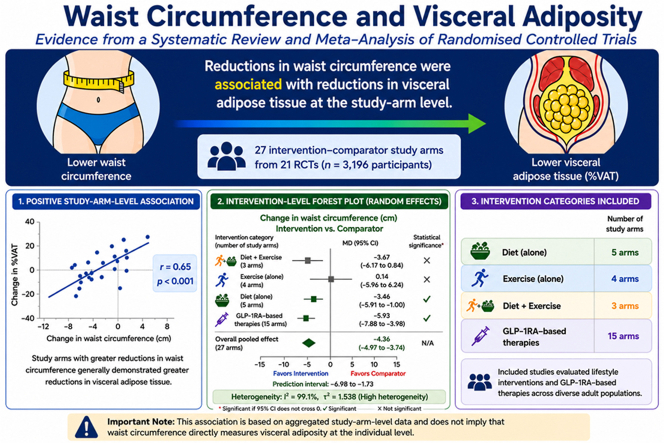

**Key Takeaway Messages**

• GLP-1RA-based therapies and dietary interventions were associated with the greatest reductions in waist circumference.
• Waist circumference change was positively associated with visceral adipose tissue change at the study-arm level.
• Baseline anthropometric characteristics and age may influence treatment response and warrant further investigation.

## 1 Introduction

### 1.1 Background and rationale

Central obesity, commonly assessed using waist circumference (WC), is strongly associated with cardiometabolic risk and increased visceral adiposity [1]. Unlike body mass index (BMI), which does not differentiate fat distribution, WC serves as a practical anthropometric marker associated with abdominal adiposity and cardiometabolic risk [2]. Randomised controlled trials (RCTs) have shown that interventions targeting energy balance and weight loss, including dietary changes, structured exercise, and pharmacotherapy, can decrease VAT and WC, thereby reducing cardiometabolic risk [3].

Among pharmacological options, glucagon-like peptide-1 receptor agonists (GLP-1RA-based therapies) have emerged as potent agents, not only for glycaemic control but also for weight reduction and VAT loss [4]. Recent large-scale trials, such as SCALE, STEP, SURMOUNT, and SELECT, confirm their efficacy in reducing body weight and enhancing cardiometabolic outcomes [[5], [6], [7], [8]]. Lifestyle-based interventions, including the Mediterranean diet, caloric restriction, and structured exercise programs, remain foundational strategies; however, their relative effects on waist circumference (WC) compared to pharmacotherapy are less well characterised. Despite the growing body of evidence, systematic comparisons of lifestyle interventions versus GLP-1RA-based therapies concerning waist circumference and visceral adipose tissue (VAT) reduction remain limited [9].

### 1.2 Knowledge gaps

Previous meta-analyses have mainly assessed weight or BMI as outcomes [10]. Few have specifically looked at WC or VAT, and variability in how results are reported (such as WC vs. imaging-based VAT, different measurement protocols, and follow-up periods) makes combined analysis difficult. Additionally, the influence of baseline factors (like age, BMI, WC, weight, and T2DM prevalence) on WC response has not been thoroughly examined across different interventions. Closing these gaps is crucial since WC reduction is increasingly seen as an important independent treatment goal.

### 1.3 Objective and PICO framework

This systematic review and meta-analysis was designed to compare the effects of lifestyle interventions (diet, exercise, or their combination) and GLP-1RA-based therapies on WC as an anthropometric correlate associated with visceral adiposity. We also aimed to identify baseline factors that may influence treatment response.

Our research question was structured using the **PICO framework**:

• **Population (P):** Adults (≥18 years) with overweight, obesity, or central adiposity, including those with or without type 2 diabetes.
• **Intervention (I):** Lifestyle-based strategies (diet, exercise, diet + exercise) or GLP-1RA-based therapies.
• **Comparator (C):** Placebo, standard care, or active comparators (e.g., alternative diet/exercise interventions, other drugs).
• **Outcome (O):** Change in WC (cm) and, where available, change in VAT (measured by imaging).

The primary pooled effect estimate was defined as the net between-group difference in change in WC from baseline to follow-up.

Eligible studies were RCTs reporting WC or VAT as primary or essential secondary outcomes in adult participants.

### 1.4 Study aim

This meta-analysis aimed to Ref. [1] synthesise evidence from RCTs reporting WC and VAT outcomes [2], compare intervention effects across lifestyle and GLP-1RA-based therapies, and [3] explore potential moderators of treatment effect, including age, BMI, WC, weight, and T2DM prevalence.

## 2 Methods

### 2.1 Study design and search strategy

This meta-analysis aimed to assess how lifestyle interventions and GLP-1RA-based therapies influence waist circumference (WC) reduction as an indicator of cardiometabolic risk, focusing on randomised controlled trials (RCTs). GLP-1RA-based therapies included both GLP-1 receptor agonists and dual incretin agonists such as tirzepatide. The search strategy targeted studies reporting either visceral adipose tissue (VAT) or WC as primary or significant secondary outcomes.

A comprehensive literature search was conducted on the PubMed database from January 2010 to August 2025, using the keywords “waist circumference,” “visceral adipose tissue,” “GLP-1 receptor agonists,” “lifestyle intervention,” “obesity,” and “randomised controlled trial.” Filters were applied to include only publications in English involving adult participants (≥18 years).

**Fig. 1:**
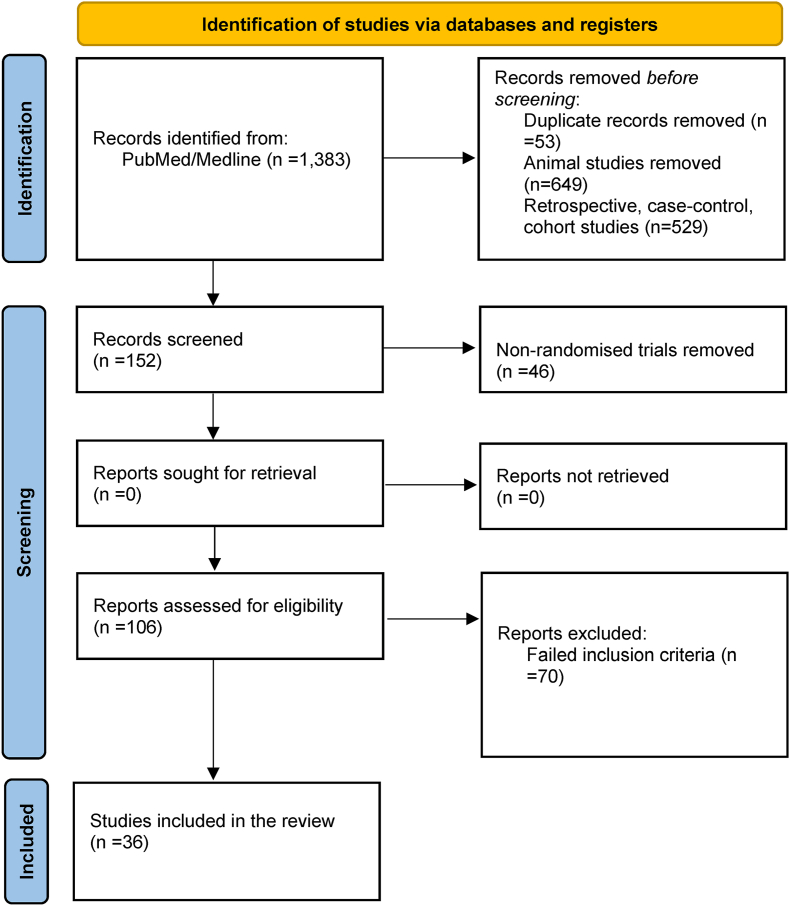
PRISMA algorithm.

The PRISMA algorithm identified 36 RCTs reporting VAT outcomes, representing the entire evidence base (Fig. 1). Of these, a subset of 21 RCTs (27 intervention-comparator study arms; n = 3196 participants) also reported WC and was therefore included in the primary analysis dataset. Interventions were categorised as exercise, diet, diet plus exercise, or GLP-1RA-based therapies. For sensitivity analyses, nine arms with active comparators (including alternative pharmacological agents or intensive lifestyle comparators such as low-calorie diets) were excluded, leaving 18 intervention-comparator study arms from 15 RCTs (n = 1524 participants) for meta-regression and subgroup analysis.

### 2.2 Registration and protocol

The protocol was registered a priori with the International Platform of Registered Systematic Review and Meta-analysis Protocols (INPLASY) under INPLASY202580071 (DOI 10.37766/inplasy2025.8.0071).

### 2.3 Data extraction and quality assessment

Data were independently extracted by two investigators (SG and AG), including study-level details such as author, year, intervention type, comparator, sample size, duration, and baseline covariates like mean age, proportion of female participants, prevalence of type 2 diabetes [T2DM], WC, body mass index [BMI], and weight.

When baseline covariates were missing in individual trials, prespecified overall means (Age: 50.3 years; T2DM prevalence: 12.5%; WC: 109.2 cm; BMI: 33.0 kg/m^2^; Weight: 93.9 kg) were used as substitutes to facilitate the calculation of pooled descriptive summaries. These imputations were applied solely for baseline descriptive reporting (Supplementary Table S1) and not for effect size calculations or meta-analyses.

Risk of bias was independently assessed by two investigators (SG and AG) using the Cochrane Risk of Bias 2 (ROB 2) tool for randomised controlled trials. The following domains were evaluated: bias arising from the randomisation process, deviations from intended interventions, missing outcome data, measurement of outcomes, and selection of reported results. Each study was classified as having low risk of bias, some concerns, or high risk of bias according to ROB 2 guidance. Discrepancies between reviewers were resolved through discussion and consensus.

### 2.4 Statistical analysis

The relationship between change in waist circumference (WC) and change in visceral adipose tissue (VAT) was assessed in the subset of study arms that reported both metrics, using Pearson's correlation coefficient (r), with scatter plots generated for visualisation.

For controlled trials, the pooled effect estimate represented the between-group mean difference (MD) in change in WC from baseline to follow-up, calculated as:

MD = (ΔWC_intervention) − (ΔWC_comparator)

Where ΔWC denotes the within-group change in waist circumference over the intervention period. Comparator groups included placebo, standard care, or active comparators, depending on the design of the individual trial.

A random-effects meta-analysis using the DerSimonian–Laird method was conducted to pool comparator-adjusted mean differences across the 27 intervention-comparator study arms primary dataset. Statistical heterogeneity was assessed using the I^2^ statistic and prediction intervals. In the event of substantial heterogeneity across studies, additional sensitivity analyses using restricted maximum likelihood (REML) estimation with a Hartung–Knapp adjustment would be conducted to assess the robustness of the pooled estimates under alternative random-effects assumptions. Sensitivity analyses were subsequently performed on the 18 intervention-comparator study arm datasets after excluding study arms with active comparators.

Subgroup analyses were performed descriptively across intervention categories (diet, exercise, diet plus exercise, and GLP-1RA-based therapies) and presented as forest plots without formal statistical comparison between subgroups. Meta-regression analyses were planned a priori to explore associations between baseline covariates (age, WC, BMI, weight, and prevalence of type 2 diabetes mellitus [T2DM]) and changes in WC. Where visual inspection or model diagnostics suggested non-linearity, higher-order terms (e.g., quadratic age terms) were evaluated to capture potential non-linear associations.

Publication bias and small-study effects were assessed using funnel plots and Egger's regression test in both the primary 27 intervention-comparator study arms dataset and the sensitivity 18 intervention-comparator study arms dataset.

All statistical analyses were conducted using R (version 4.4.3; R Foundation for Statistical Computing, Vienna, Austria), employing the meta and metafor packages for meta-analysis. Figures were generated primarily in R using ggplot2, while the correlation scatterplot was created in Python (version 3.11.9) using matplotlib (version 3.9.2). Results were reported with 95% confidence intervals (CI), and statistical significance was defined as p < 0.05. Forest plots included pooled effects with prediction intervals, whereas bubble plots displayed relationships between significant moderators and WC change weighted by the inverse of the standard error.

All R and Python scripts, together with the master dataset used for analyses, are publicly available via Figshare (https://doi.org/10.6084/m9.figshare.29974606.v1; Ghosal & Ghosal, 2025).

This systematic review and meta-analysis was conducted and reported in accordance with the PRISMA 2020 statement and checklist (Supplementary Appendix S1).

## 3 Results

### 3.1 Study characteristics

The meta-analysis included 36 randomised controlled trials (RCTs) published between 2012 and 2025, involving 6581 participants across 49 intervention-comparator study arms, all reporting visceral adipose tissue (VAT) as an outcome [[11], [12], [13], [14], [15], [16], [17], [18], [19], [20], [21], [22], [23], [24], [25], [26], [27], [28], [29], [30], [31], [32], [33], [34], [35], [36], [37], [38], [39], [40], [41], [42], [43], [44], [45], [46]]. Among these, 21 RCTs (27 intervention-comparator study arms; n = 3196 participants) also reported waist circumference (WC) and were part of the primary analysis (Table 1). These 27 intervention-comparator study arms consisted of interventions such as exercise (4 arms), diet alone (5 arms), diet plus exercise (3 arms), and GLP-1RA-based therapies (15 arms). A subsequent sensitivity analysis excluded nine arms with active comparators, resulting in 18 intervention-comparator study arms from 15 RCTs (n = 1524 participants) for meta-regression.

**Table 1:** **Characteristics of included randomised controlled trials evaluating lifestyle and GLP-1RA-based interventions on waist circumference (WC) and visceral adipose tissue (VAT).** Studies are grouped according to intervention modality, including diet-based interventions, exercise-based interventions, combined diet-plus-exercise interventions, and GLP-1 receptor agonist–based therapies. WC denotes waist circumference; VAT denotes visceral adipose tissue assessed using imaging-based methods (MRI, CT, or DXA-derived estimates where applicable).

Study	Population	Intervention Category	Intervention	Comparator	Duration	WC	VAT
**Diet-Based Interventions**							
Bajerska et al. [12]	Centrally obese postmenopausal women with metabolic syndrome features	Diet	Mediterranean or Central European high-fibre diet	Active comparator (dietary)	16 weeks	Yes	Yes
George et al. [16]	Adults with NAFLD and insulin resistance	Diet	Mediterranean diet	Active comparator (Low-fat diet)	12 weeks	Yes	Yes
Moreno et al. [20]	Adults with obesity	Diet	Very low-calorie ketogenic diet	Active comparator (low-calorie diet)	24 months	Yes	Yes
Porca et al. [21]	Adults with obesity	Diet	Intensive educational Mediterranean-pattern dietary intervention	Usual care	12 months	Yes	Yes
van Gemert et al. [31]	Overweight postmenopausal women	Diet; Diet + Exercise	Calorie-restricted diet; exercise plus mild caloric restriction	Usual care	16 weeks	Yes	Yes
**Exercise-Based Interventions**							
Pedersen et al. [26]	Overweight/pre-diabetic patients with coronary artery disease	Exercise	Aerobic interval training	Active comparator (Low-energy diet)	12 weeks	Yes	Yes
Polo-Ferrero et al. [30]	Older women with sarcopenic obesity	Exercise	Power training/multicomponent training	Usual care	32 weeks	Yes	Yes
Barbosa et al. [11]	High cardiovascular-risk adults aged ≥50 years	Exercise; Diet + Exercise	Supervised exercise; Mediterranean-inspired diet plus exercise	Usual care	12 weeks	Yes	Yes
Miralles-Llumà et al. [19]	Adults with severe obesity and obstructive sleep apnea	Diet + Exercise	Hypocaloric Mediterranean diet plus exercise	Usual care	12 months	Yes	Yes
**GLP-1-Based Therapy Studies: Liraglutide**							
Astrup et al. [41]	Obese non-diabetic adults	GLP-1 receptor agonist	Liraglutide 1.2–3.0 mg/day	Active comparator (orlistat)	2 years	Yes	Yes
Bizino et al. [34]	Adults with T2DM and BMI >25 kg/m^2^	GLP-1 receptor agonist	Liraglutide 1.8 mg/day	Placebo	26 weeks	Yes	Yes
Frossing et al. [38]	Women with PCOS and BMI >25 kg/m^2^	GLP-1 receptor agonist	Liraglutide 1.8 mg/day	Placebo	26 weeks	Yes	Yes
Larsen et al. [39]	Patients with schizophrenia spectrum disorder and prediabetes	GLP-1 receptor agonist	Liraglutide 1.8 mg/day	Placebo	16 weeks	Yes	Yes
Neeland et al. [35]	Adults with overweight/obesity at high CV risk without T2DM	GLP-1 receptor agonist	Liraglutide 3.0 mg/day	Placebo	40 weeks	Yes	Yes
Santilli et al. [33]	Obese adults with prediabetes or early T2DM	GLP-1 receptor agonist	Liraglutide 1.8 mg/day	Active comparator (Lifestyle counselling)	∼4 months	No	Yes
van Eyk et al. [36]	South Asian patients with T2DM	GLP-1 receptor agonist	Liraglutide 1.8 mg/day	Placebo	26 weeks	Yes	Yes
Yu et al. [40]	Adults with obesity and T2DM	GLP-1 receptor agonist	Liraglutide + background antidiabetic therapy	Active comparator (Lifestyle intervention)	12 weeks	Yes	Yes
**GLP-1-Based Therapy Studies: Semaglutide**							
Kadowaki et al. [43]	East Asian adults with obesity ± T2DM	GLP-1 receptor agonist	Semaglutide 1.7/2.4 mg weekly	Placebo	68 weeks	Yes	Yes
McCrimmon et al. [42]	Adults with uncontrolled T2DM on metformin	GLP-1 receptor agonist	Semaglutide 1.0 mg weekly	Active comparator (Canagliflozin)	52 weeks	No	Yes
Wilding et al. [44]	Adults with overweight/obesity without diabetes	GLP-1 receptor agonist	Semaglutide 2.4 mg weekly	Placebo	68 weeks	Yes	Yes
**Dual GIP/GLP-1 Agonist Study**							
Gastaldelli et al. (SURPASS-3) [45]	Adults with T2DM and fatty liver	Dual GIP/GLP-1 receptor agonist	Tirzepatide 5/10/15 mg weekly	Active comparator (Insulin degludec)	52 weeks	Yes	Yes

Baseline characteristics across the 27 intervention-comparator study arms WC dataset showed a weighted mean age of 55.2 years, with 63.8% female participants and a 28.0% prevalence of type 2 diabetes. Anthropometric baselines included a mean waist circumference of 107.9 cm, a BMI of 33.9 kg/m^2^, and a weight of 92.8 kg. Detailed intervention-specific baseline characteristics are summarised in Supplementary Table S1, while the complete study-level dataset is available in the Figshare data repository.

### 3.2 Correlation between WC and %VAT change

**Fig. 2:**
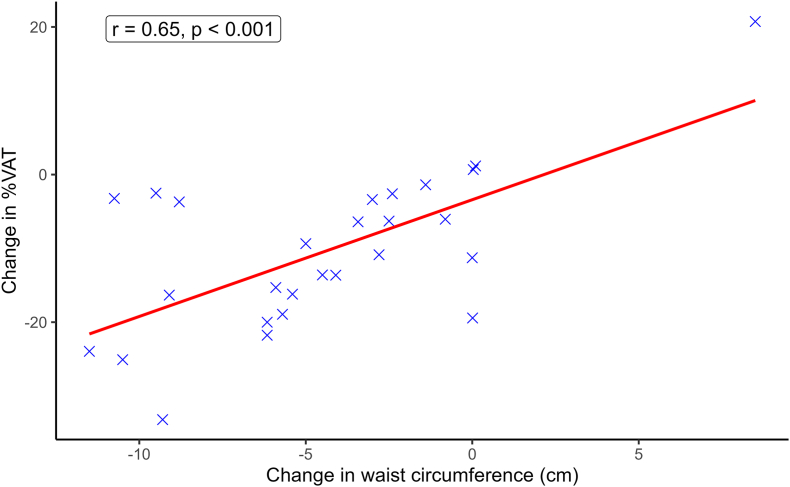
Correlation between change in waist circumference (ΔWC) and percentage change in visceral adipose tissue (Δ%VAT) across 27 intervention-comparator study arms (21 RCTs). Each point represents one study arm.

Across the 27 intervention-comparator study arms reporting both waist circumference and visceral adipose tissue outcomes, a strong positive correlation was observed between reductions in WC and %VAT change (r = 0.65, 95% CI 0.36–0.83; p < 0.001) (Fig. 2). This suggests that WC change is associated with VAT change at the study-arm level and may represent a practical population-level anthropometric correlate in intervention trials. A sensitivity analysis excluding study arms with upper-quartile standard errors further strengthened the correlation (r = 0.84, 95% CI 0.60–0.94; p < 0.001; n = 16). (Supplementary Figure S1).

### 3.3 Primary intervention impact analysis

**Fig. 3:**
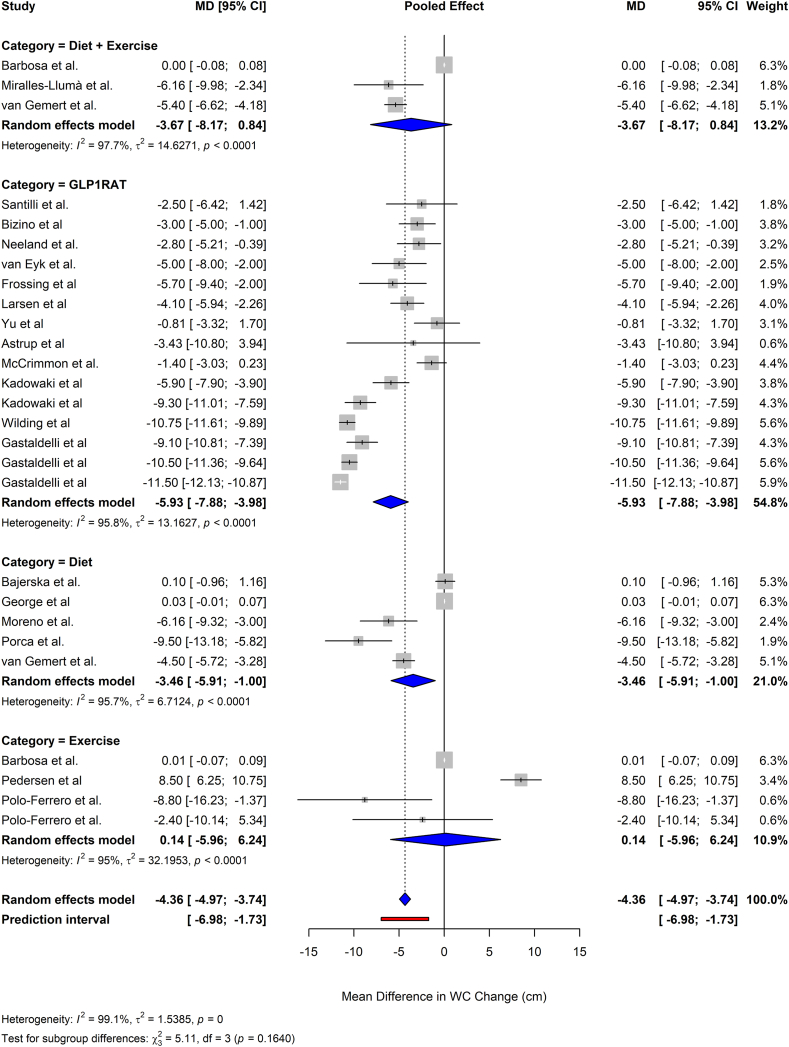
Forest plot of mean differences in change in waist circumference (WC) change (cm) across 27 intervention-comparator study arms (21 RCTs), stratified by intervention category. Pooled effects were calculated using a random-effects model (DerSimonian–Laird), with heterogeneity expressed as prediction intervals.

In the primary meta-analysis of 27 intervention-comparator study arms (21 RCTs), compared with comparator groups, interventions were associated with significant reductions in waist circumference. The pooled mean difference was −4.36 cm (95% CI -4.97 to −3.74; prediction interval −6.98 to −1.73) (Fig. 3).

### 3.4 By intervention category

• **Diet plus Exercise (3 arms)**: Mean reduction −3.67 cm (95% CI -8.17 to 0.84, PI -12.41 to 5.08), with substantial heterogeneity.
• **GLP-1RA-based therapies (15 arms)**: Consistent reduction −5.93 cm (95% CI -7.88 to −3.98, PI -13.58 to 1.81); heterogeneity remained high.
• **Diet alone (5 arms)**: Reduction −3.46 cm (95% CI -5.91 to −1.00, PI -7.03 to 1.62); heterogeneity high.
• **Exercise alone (4 arms)**: Non-significant pooled effect 0.14 cm (95% CI -5.96 to 6.24, PI -12.54 to 12.83); heterogeneity high.

The test for subgroup differences was not statistically significant (p = 0.16), suggesting no clear evidence that one intervention category was superior in reducing WC.

Because substantial heterogeneity was observed across analyses, an additional sensitivity analysis was performed using restricted maximum likelihood (REML) estimation with a Hartung–Knapp adjustment. The pooled effect estimate remained statistically significant and directionally consistent with the primary DerSimonian–Laird analysis (MD −4.41 cm; 95% CI −6.20 to −2.62) (Supplementary Figure S2). However, confidence intervals widened modestly under the more conservative variance estimation approach. Prediction interval analysis demonstrated substantial between-study variability (−13.66 to 4.83 cm), indicating that future studies may show heterogeneous effect sizes. Subgroup differences remained statistically non-significant (p = 0.277).

### 3.5 Sensitivity analysis

**Fig. 4:**
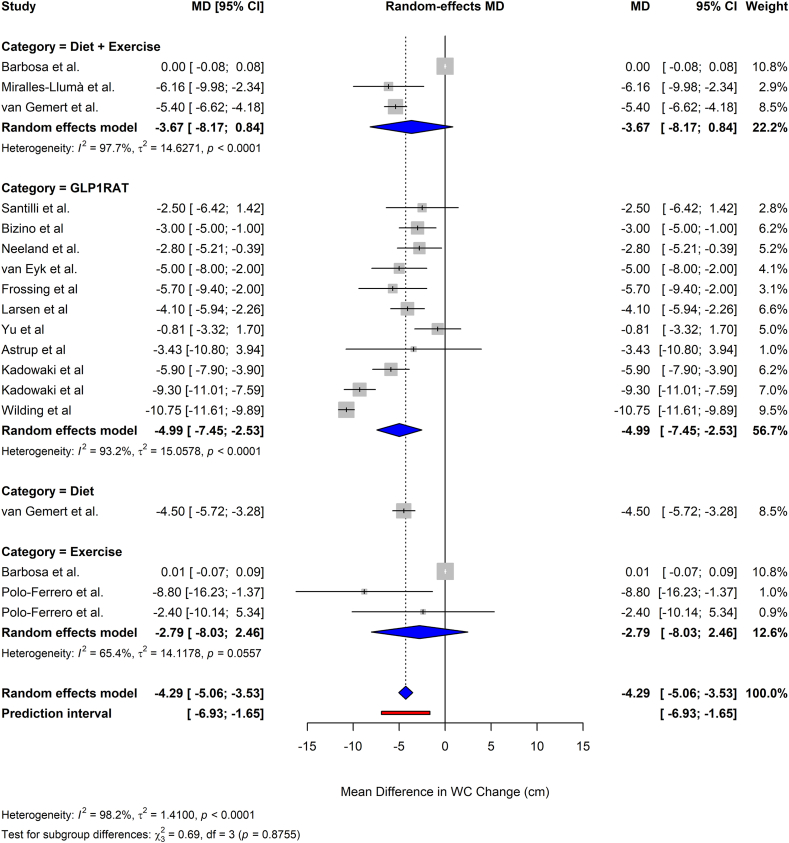
Sensitivity analysis forest plot of mean difference in waist circumference (WC) change (cm) across 18 intervention-comparator study arms (15 RCTs), after excluding nine arms with active comparators. Pooled effects were calculated using a random-effects model (DerSimonian–Laird), with heterogeneity expressed as prediction intervals.

Excluding the nine arms with active comparators reduced the dataset to 18 intervention-comparator study arms from 15 RCTs (n = 1524 participants). The pooled analysis remained consistent with the primary findings, demonstrating a significant reduction in waist circumference of −4.29 cm (95% CI -5.06 to −3.53; prediction interval −6.93 to −1.65) (Fig. 4).

### 3.6 By intervention category

• **Diet plus Exercise (2 arms):** Pooled reduction −3.67 cm (95% CI -8.17 to 0.84), with high heterogeneity (I^2^ = 97.7%).
• **GLP-1RA-based therapies (11 arms):** Pooled reduction −4.99 cm (95% CI -7.45 to −2.53), with high heterogeneity (I^2^ = 93.2%).
• **Diet alone (1 arm):** Reduction −4.50 cm (95% CI -5.72 to −3.28).
• **Exercise alone (4 arms):** Non-significant pooled reduction −2.79 cm (95% CI-8.03 to 2.46); moderate heterogeneity (I^2^ = 65.4%).

The overall heterogeneity remained high (Prediction Interval: −6.93 to −1.65), but the effect estimates were robust after excluding active comparator arms. The test for subgroup differences remained non-significant (p = 0.88).

### 3.7 Exploratory analysis (meta-regression & small-study effects)

Exploratory meta-regression analyses were performed to examine potential moderators within the 18 intervention-comparator study arm dataset, as planned. The quadratic term for age (Age^2^) was highly significant (β = 0.0021, SE = 0.0002, 95% CI [0.0018, 0.0025], p < 0.0001), accounting for 77.7% of heterogeneity, which indicates a non-linear effect across the age range (∼42–70 years). T2DM prevalence was not significant (β = 0.0116, SE = 0.0171, 95% CI [−0.0218, 0.0451], p = 0.496, R^2^ = 3.1%). Baseline WC (β = −0.308, SE = 0.111, 95% CI [−0.526, −0.090], p = 0.006, R^2^ = 34.1%), baseline BMI (β = −0.865, SE = 0.073, 95% CI [−1.007, −0.723], p < 0.0001, R^2^ = 79.4%), and baseline weight (β = −0.216, SE = 0.016, 95% CI [−0.248, −0.185], p < 0.0001, R^2^ = 83.2%) emerged as significant moderators, with baseline weight explaining the most heterogeneity. Baseline weight showed the strongest exploratory association with WC reduction among the evaluated moderators (R^2^ analogue: 83.2%), although these findings should be interpreted with caution given the limited number of study arms and potential collinearity among anthropometric variables. Meta-regression results are detailed in Supplementary Table S2. Bubble plots for the significant moderators (baseline WC, baseline BMI, baseline weight) showed inverse relationships with WC change, whereas baseline age demonstrated a quadratic association (Supplementary Figures S3).

Assessment of small-study effects indicated evidence of asymmetry. Funnel plots of the primary 27 intervention-comparator study arms dataset showed visual asymmetry, with Egger's regression test confirming statistical significance (t = −3.36, df = 25, p = 0.0025). This asymmetry persisted in the sensitivity dataset restricted to placebo- or lifestyle-controlled arms (18 arms; t = −3.38, df = 16, p = 0.0038), suggesting that small-study effects were not solely due to active comparator trials (Supplementary Figure S4). The ROB 2 assessment showed that most trials had a low risk of bias across domains, with only two studies raising some concerns related to missing outcome data. Domain-level and overall ROB 2 assessments are presented in Supplementary Figure S5.

## 4 Discussion

### 4.1 Principal findings

In this meta-analysis of 21 randomised controlled trials (27 intervention-comparator study arms), we found that interventions targeting lifestyle modification (diet, exercise, or their combination) and GLP-1RA-based therapies were associated with significant reductions in change in waist circumference (WC), a clinically relevant anthropometric marker associated with visceral adiposity and cardiometabolic risk. GLP-1RA-based therapies and diet-based interventions demonstrated statistically significant reductions, whereas exercise-only and diet-plus-exercise interventions showed more variable effects. Baseline BMI, body weight, and WC were strong moderators of the observed intervention effects, with higher baseline values predicting greater reductions in WC. Importantly, pooled effect estimates represented net between-group differences in WC change between intervention and comparator arms rather than within-arm pre–post changes alone. Additionally, age exhibited a non-linear (quadratic) association with WC response, highlighting the potential influence of life-course factors on visceral fat responsiveness to interventions. Funnel plot asymmetry and Egger's regression indicated potential small-study effects, suggesting caution in interpreting absolute effect sizes. Overall, the findings emphasise the robustness of intervention effects on WC while underscoring heterogeneity driven by baseline characteristics.

### 4.2 Comparison with previous literature

Our results are consistent with prior evidence that GLP-1RA-based therapies achieve significant reductions in visceral adiposity and WC beyond the effects of weight loss alone [47]. The strong association between baseline body weight and WC response aligns with large-scale trials showing that individuals with higher adiposity derive greater relative and absolute benefit [48]. The non-linear association with age observed here resonates with emerging evidence that metabolic responsiveness may attenuate in older populations due to sarcopenia, altered fat distribution, and endocrine changes [49].

In contrast, exercise-only interventions demonstrated less consistent effects on WC reduction, which is in line with reports suggesting that structured exercise preferentially improves metabolic fitness rather than substantially altering central adiposity unless combined with dietary restriction [50]. Diet interventions, particularly calorie-restricted and Mediterranean-based approaches, replicated the well-established effect of energy restriction on central fat reduction.

### 4.3 Clinical implications

The findings carry several practical implications. First, WC should be considered a vital target outcome in intervention studies, given its established association with cardiovascular, renal, and metabolic disease beyond BMI. Second, GLP-1RA-based therapies were associated with the largest reductions in WC within subgroup analyses, although formal subgroup differences were not statistically significant. Third, the observed effect modification by baseline anthropometric measures suggests that tailoring interventions to individual risk profiles (e.g., higher BMI or WC at baseline) may optimise treatment response. The quadratic relationship with age highlights the importance of early intervention in mid-life before responsiveness diminishes.

### 4.4 Strengths and limitations

This study has several strengths, including comprehensive identification of RCTs, use of robust random-effects models with prediction intervals, and systematic exploration of heterogeneity via meta-regression. The inclusion of both pharmacological and lifestyle interventions allows for cross-comparison across intervention modalities. Additionally, the focus on WC as a surrogate marker for visceral adiposity provides clinically meaningful insight into central fat dynamics.

However, limitations must be acknowledged. First, the observed relationship between WC change and VAT change was derived from aggregated study-arm-level data rather than individual participant-level analyses. Therefore, these findings should not be interpreted as validating WC as a direct individual-level surrogate for VAT reduction. Imaging-based VAT measures were also inconsistently reported across studies. Second, heterogeneity across interventions (e.g., differing exercise protocols, dietary compositions, and drug doses) may limit direct comparability. The persistently high heterogeneity across intervention categories, including prediction intervals that cross the null despite significant pooled estimates, suggests considerable between-study variability and indicates that pooled subgroup estimates should be interpreted with caution. Third, evidence of small-study effects suggests that intervention efficacy could be overestimated. Fourth, the exploratory meta-regression analyses should be interpreted cautiously. The number of intervention-comparator study arms available for meta-regression was limited, reducing statistical power for moderator analyses. In addition, baseline anthropometric variables such as BMI, waist circumference, and body weight are biologically correlated, which may introduce collinearity and unstable moderator estimates. Consequently, these findings should be considered hypothesis-generating rather than definitive evidence of predictive relationships. Additionally, the literature search was restricted to PubMed/MEDLINE and English-language publications, which may have resulted in the omission of potentially relevant studies indexed exclusively in other databases. Finally, although the risk of bias was generally low, a small number of studies had some concerns regarding missing outcome data, which could introduce bias in pooled estimates.

### 4.5 Future directions

Future studies should incorporate standardised WC and imaging-based VAT outcomes to allow harmonisation across trials. Longitudinal RCTs with extended follow-up are needed to determine whether WC reductions translate consistently into improvements in hard outcomes such as cardiovascular events, renal progression, or mortality. Exploration of age-related non-linear responses warrants mechanistic studies into adipose tissue biology across the life course. Additionally, trials examining combination strategies (GLP-1RA-based therapies with structured lifestyle interventions) may provide insight into whether synergistic effects can be achieved. Individual participant-level analyses examining the relationship between WC and imaging-based VAT changes are needed to clarify the extent to which WC reflects changes in visceral versus subcutaneous abdominal adiposity.

## 5 Conclusion

This meta-analysis suggests that GLP-1RA-based therapies and dietary interventions are associated with reductions in waist circumference, while intervention effects varied across exercise-based strategies. Baseline anthropometric characteristics and age appeared to modulate the extent of response. WC reduction should be considered a key target in interventions aimed at mitigating cardiometabolic risk, and future research should focus on harmonised measurement and mechanistic understanding of modifiers of treatment response.

## Author contributions

Samit Ghosal: Conceptualisation; Methodology; Data curation; Formal analysis; Writing – original draft; Visualisation; Supervision. Anuradha Ghosal: Literature search; Screening and eligibility assessment; Data extraction; Validation; Writing – review & editing. Both authors approved the final manuscript.

## Declaration of artificial intelligence

An artificial intelligence-assisted tool (Grammarly) was used during manuscript preparation for language refinement and correction of grammatical errors only. All scientific interpretation, data verification, statistical decisions, and final manuscript content were independently conducted, reviewed and approved by the authors. The graphical abstract was prepared using notebooklm.

## Funding

This research did not receive any specific grant from funding agencies in the public, commercial, or not-for-profit sectors.

## Conflicts of interest

The authors declare no conflicts of interest.

**Registration**: INPLASY202580071; https://doi.org/10.37766/inplasy2025.8.0071**.**

**Data Availability:** All raw data and reproducible code are openly available via Figshare at: https://doi.org/10.6084/m9.figshare.29974606.v1 (Ghosal & Ghosal, 2025).
