# Supplementary material for: Effects of lifestyle and glucagon-like Peptide-1 receptor agonist-based therapies on waist circumference: A systematic review and meta-analysis
Source: Obes Pillars. 2026 May 30;19:100281. doi: 10.1016/j.obpill.2026.100281 (PMC13272577; doi:10.1016/j.obpill.2026.100281)

**Supplementary Appendix**

**Supplementary Appendix S1: The PRISMA checklist**

| Section & Topic | Item | PRISMA 2020 Checklist Item | Location in Manuscript |
| --- | --- | --- | --- |
| TITLE | 1 | Identify the report as a systematic review/meta-analysis | Title Page |
| ABSTRACT | 2 | See PRISMA 2020 for Abstracts checklist | Summary/Abstract, Pages 2 |
| INTRODUCTION | 3 | Describe rationale for the review | Introduction: Background and Rationale |
| INTRODUCTION | 4 | Provide explicit statement of objectives/questions | Objective and PICO Framework |
| METHODS | 5 | Specify inclusion and exclusion criteria | Methods: Study Design and Search Strategy |
| METHODS | 6 | Specify information sources searched | Methods: Study Design and Search Strategy |
| METHODS | 7 | Present full search strategy | Methods + Supplementary Material |
| METHODS | 8 | Specify selection process | PRISMA flow diagram + Methods |
| METHODS | 9 | Specify data collection process | Data Extraction and Quality Assessment |
| METHODS | 10a | List and define outcomes sought | Objective and PICO Framework |
| METHODS | 10b | List other variables sought | Data Extraction and Quality Assessment |
| METHODS | 11 | Specify risk of bias assessment methods | Risk of Bias section |
| METHODS | 12 | Specify effect measures used | Statistical Analysis |
| METHODS | 13a | Describe synthesis process | Statistical Analysis |
| METHODS | 13b | Methods to prepare data | Statistical Analysis |
| METHODS | 13c | Methods to tabulate/display results | Statistical Analysis |
| METHODS | 13d | Describe synthesis methods | Statistical Analysis |
| METHODS | 13e | Methods to explore heterogeneity | Meta-regression & subgroup analysis |
| METHODS | 13f | Sensitivity analyses | Sensitivity Analysis section |
| METHODS | 14 | Describe reporting bias assessment | Statistical Analysis |
| METHODS | 15 | Describe certainty assessment | Not formally performed |
| RESULTS | 16a | Describe search and selection results | PRISMA Figure 1 |
| RESULTS | 16b | Cite excluded studies/reasons | PRISMA Figure 1 |
| RESULTS | 17 | Cite included studies | Table 1 |
| RESULTS | 18 | Present risk of bias results | ROB2 results + Supplementary Figure S5 |
| RESULTS | 19 | Present results of individual studies | Figures 3 and 4; Table 1 |
| RESULTS | 20a | Summarise characteristics and risk of bias | Results section |
| RESULTS | 20b | Present pooled synthesis results | Figures 3 and 4 |
| RESULTS | 20c | Present heterogeneity investigations | Meta-regression section |
| RESULTS | 20d | Present sensitivity analyses | Sensitivity Analysis |
| RESULTS | 21 | Present reporting bias assessment | Funnel plot & Egger’s test |
| RESULTS | 22 | Present certainty of evidence assessment | Not formally performed |
| DISCUSSION | 23a | General interpretation of findings | Discussion: Principal Findings |
| DISCUSSION | 23b | Discuss limitations of evidence | Strengths and Limitations |
| DISCUSSION | 23c | Discuss limitations of review process | Strengths and Limitations |
| DISCUSSION | 23d | Implications for practice/research | Clinical Implications + Future Directions |
| OTHER INFORMATION | 24a | Registration information | Registration section |
| OTHER INFORMATION | 24b | Protocol access | Registration section |
| OTHER INFORMATION | 24c | Amendments to protocol | None |
| OTHER INFORMATION | 25 | Sources of support | Funding |
| OTHER INFORMATION | 26 | Competing interests | Conflicts of Interest |
| OTHER INFORMATION | 27 | Availability of data/materials | Data Availability |

**Supplementary Table S1: Baseline characteristics of included study arms (27 arms, 21 RCTs).**Values are reported as sample-size–weighted means within each intervention category. *Female (%) ** represents the weighted proportion of female participants, and *T2DM (%) ** represents the weighted proportion of participants with type 2 diabetes across study arms. When baseline values were not available, imputed means were applied according to the prespecified strategy (Age: 50.3 years; T2DM prevalence: 12.5%; WC: 109.2 cm; BMI: 33.0 kg/m²; Weight: 93.9 kg). VAT values were omitted due to heterogeneous reporting units (cm², litres, %, kg); the complete study-level dataset, including VAT where reported, is provided in the supplementary repository.

| Category | Arms (n) | Participants (N) | Mean Age (yrs) | Female (%) * | T2DM (%) * | WC (cm) | BMI (kg/m²) | Weight (kg) |
| --- | --- | --- | --- | --- | --- | --- | --- | --- |
| Exercise | 4 | 191 | 69.2 | 59.1 | 0.0 | 109.5 | 30.8 | 80.8 |
| Diet | 5 | 808 | 53.9 | 88.3 | 4.6 | 108.2 | 36.0 | 96.9 |
| Diet + Exercise | 3 | 256 | 60.6 | 79.0 | 0.7 | 100.1 | 29.7 | 80.6 |
| GLP1RAT | 15 | 1,941 | 51.6 | 54.3 | 48.9 | 108.3 | 33.4 | 94.1 |
| Overall | 27 | 3,196 | 55.2 | 63.8 | 28.0 | 107.9 | 33.9 | 92.8 |

**Supplementary Table S2:** Exploratory meta-regression analyses evaluating potential moderators of waist circumference change in the 18-arm sensitivity dataset. Results are presented as regression coefficients (β) with standard error (SE), 95% confidence interval (CI), p-values, and analogue R² (% of between-study heterogeneity explained). *The quadratic term for age (Age²) was included after evidence of a non-linear effect across the observed age range (~42–70 years).*

| Moderator | Beta | SE | 95% CI | p-value | R² analogue (%) |
| --- | --- | --- | --- | --- | --- |
| Age² (years²) | 0.0021 | 0.0002 | 0.0018 to 0.0025 | <0.0001 | 77.73 |
| T2DM prevalence (%) | 0.0116 | 0.0171 | −0.0218 to 0.0451 | 0.496 | 3.10 |
| Baseline WC (cm) | −0.3078 | 0.1113 | −0.5259 to −0.0897 | 0.0057 | 34.09 |
| Baseline BMI (kg/m²) | −0.8647 | 0.0726 | −1.0069 to −0.7225 | <0.0001 | 79.44 |
| Baseline Weight (kg) | −0.2163 | 0.0162 | −0.2479 to −0.1846 | <0.0001 | 83.18 |

**Supplementary Figure S1:** Sensitivity analysis scatter plot of change in waist circumference (ΔWC) versus percentage change in visceral adipose tissue (Δ%VAT) across 16 study arms, excluding those with upper-quartile standard errors.


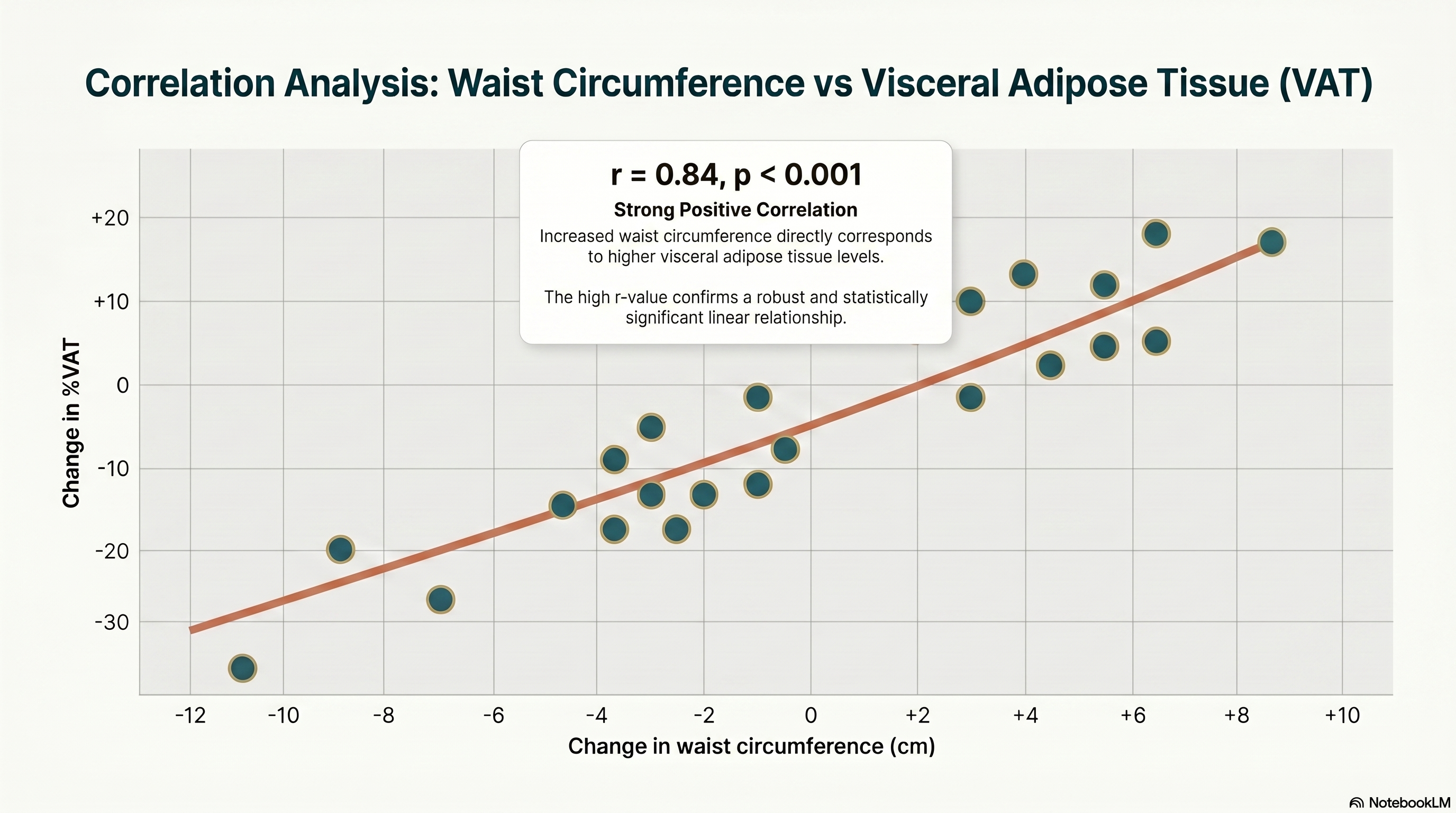


**Supplementary Figure S2:** Sensitivity Forest plot using restricted maximum likelihood (REML) estimation with Hartung–Knapp adjustment for pooled mean difference (MD) in waist circumference (WC) change across intervention categories. Squares represent study-specific effect estimates weighted by inverse variance, with horizontal lines indicating 95% confidence intervals (CI). Diamonds represent pooled subgroup and overall random-effects estimates. Prediction intervals are shown for the overall pooled analysis. Heterogeneity statistics (I² and τ²) are displayed for each subgroup and the overall model. Subgroup differences were not statistically significant (p = 0.277).


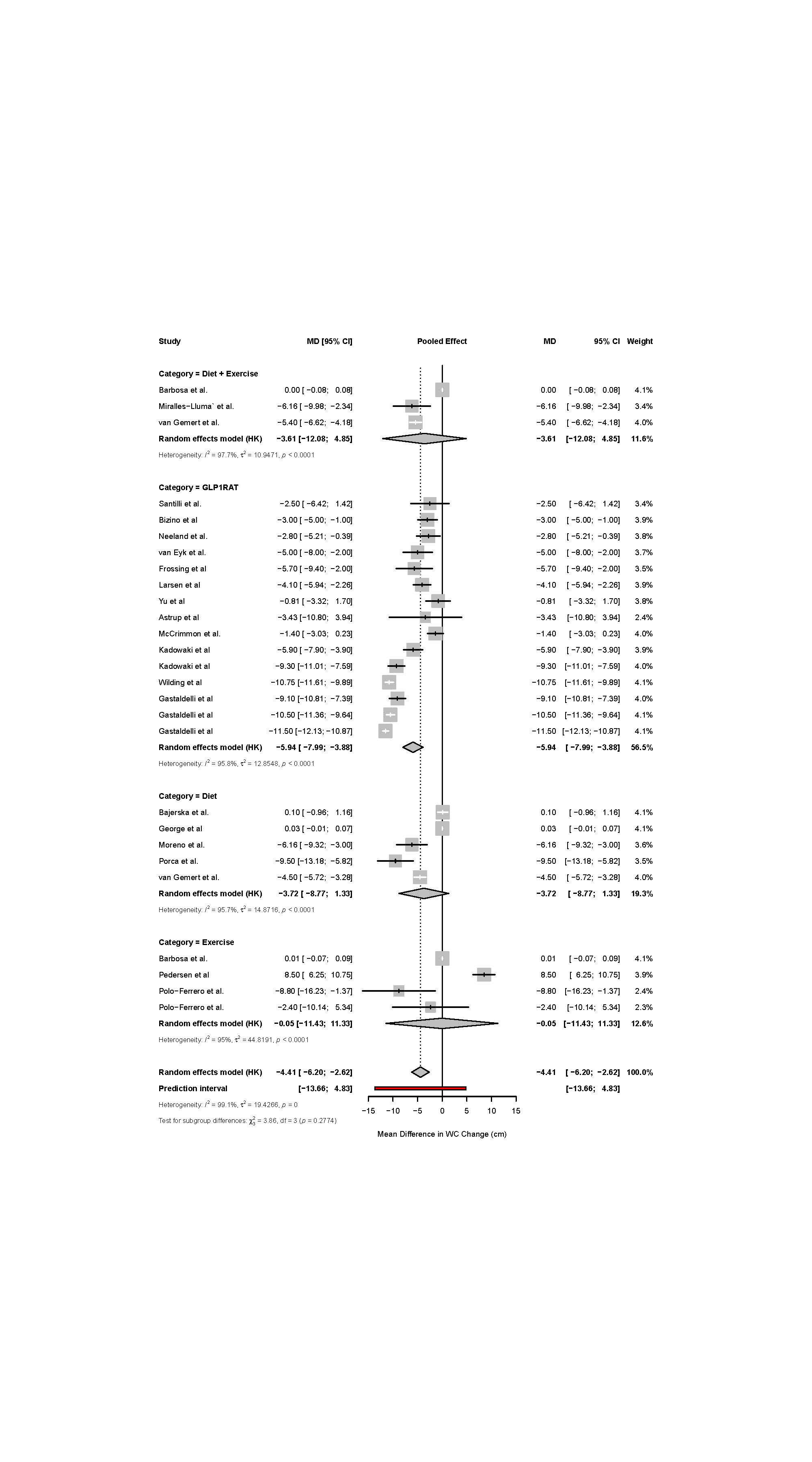


**Supplementary Figure S3:** Bubble plots of baseline moderators versus change in waist circumference (WC) across the 18-arm sensitivity dataset.
(A) Baseline age (quadratic fit), (B) baseline BMI, (C) baseline waist circumference, and (D) baseline body weight. Each circle represents an individual study arm, with bubble size weighted by inverse variance (larger bubbles indicate greater precision). Solid lines represent fitted regression relationships (quadratic for age; linear for BMI, WC, and weight) with shaded 95% confidence bands.


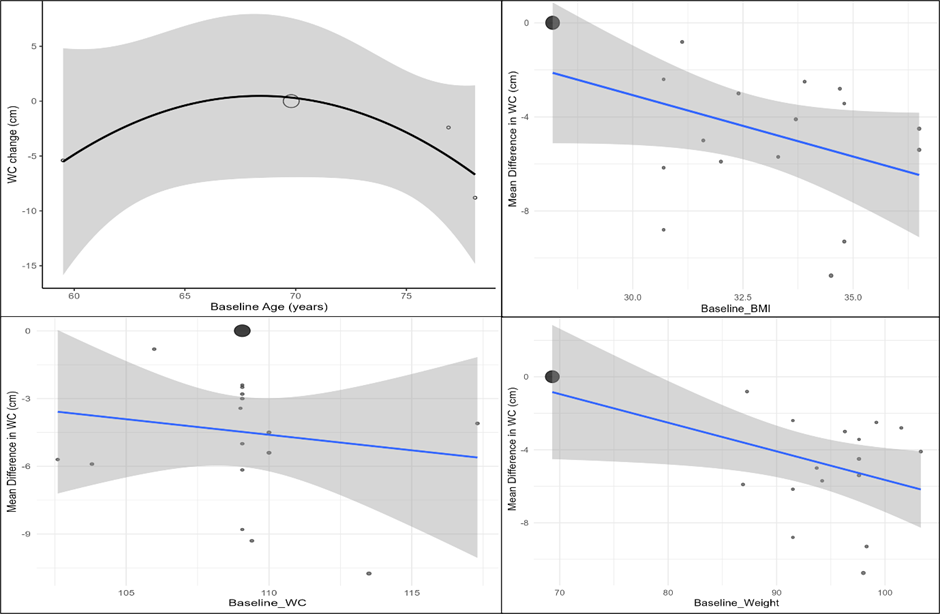


**Supplementary Figure S4:** (A) Funnel plot of the 27-arm primary dataset. (B) Funnel plot of the 18-arm sensitivity dataset restricted to placebo- or lifestyle-controlled arms. Each circle represents a study arm, plotted by effect size (mean difference in waist circumference [WC] change, cm) against its standard error. The dashed vertical line indicates the pooled fixed-effect estimate, while the sloping dashed lines represent pseudo 95% confidence limits. Egger’s regression test demonstrated significant funnel plot asymmetry in both analyses (primary: *p* = 0.0025; sensitivity: *p* = 0.0038).


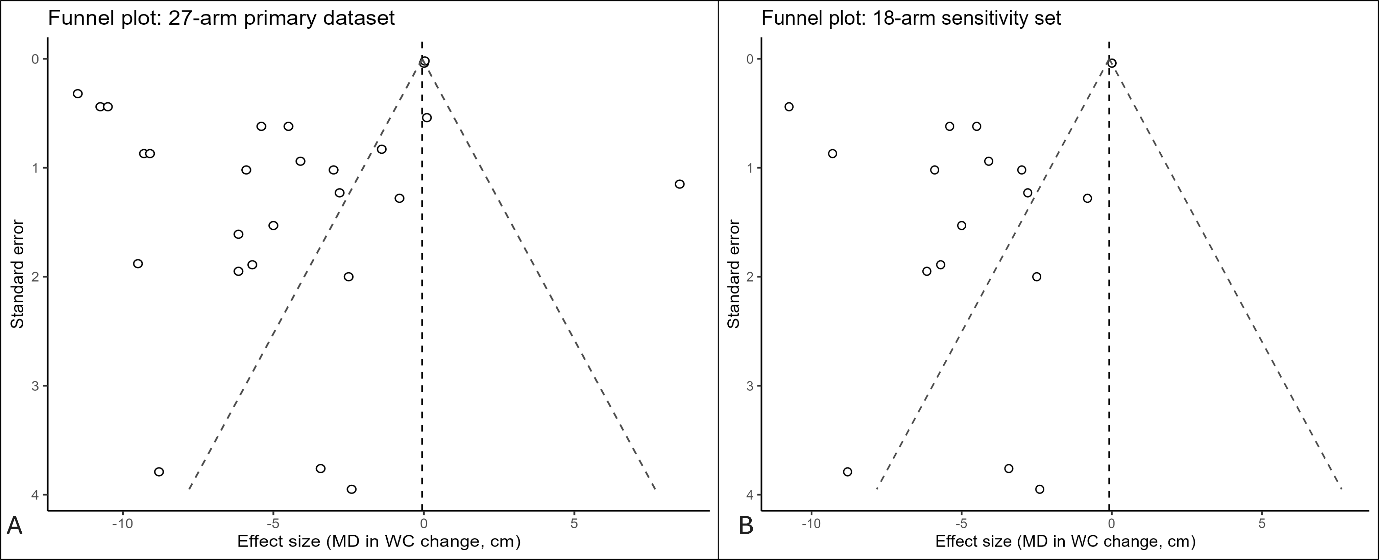


**Supplementary Figure S5. Risk-of-bias assessment using the Cochrane Risk of Bias 2 (ROB 2) tool for randomised controlled trials.**
Traffic-light plots show individual study-level assessments across five ROB 2 domains: D1, bias arising from the randomisation process; D2, bias due to deviations from intended interventions; D3, bias due to missing outcome data; D4, bias in measurement of outcomes; and D5, bias in selection of the reported result. The summary plot presents the proportion of studies rated as low risk or some concerns across each domain.


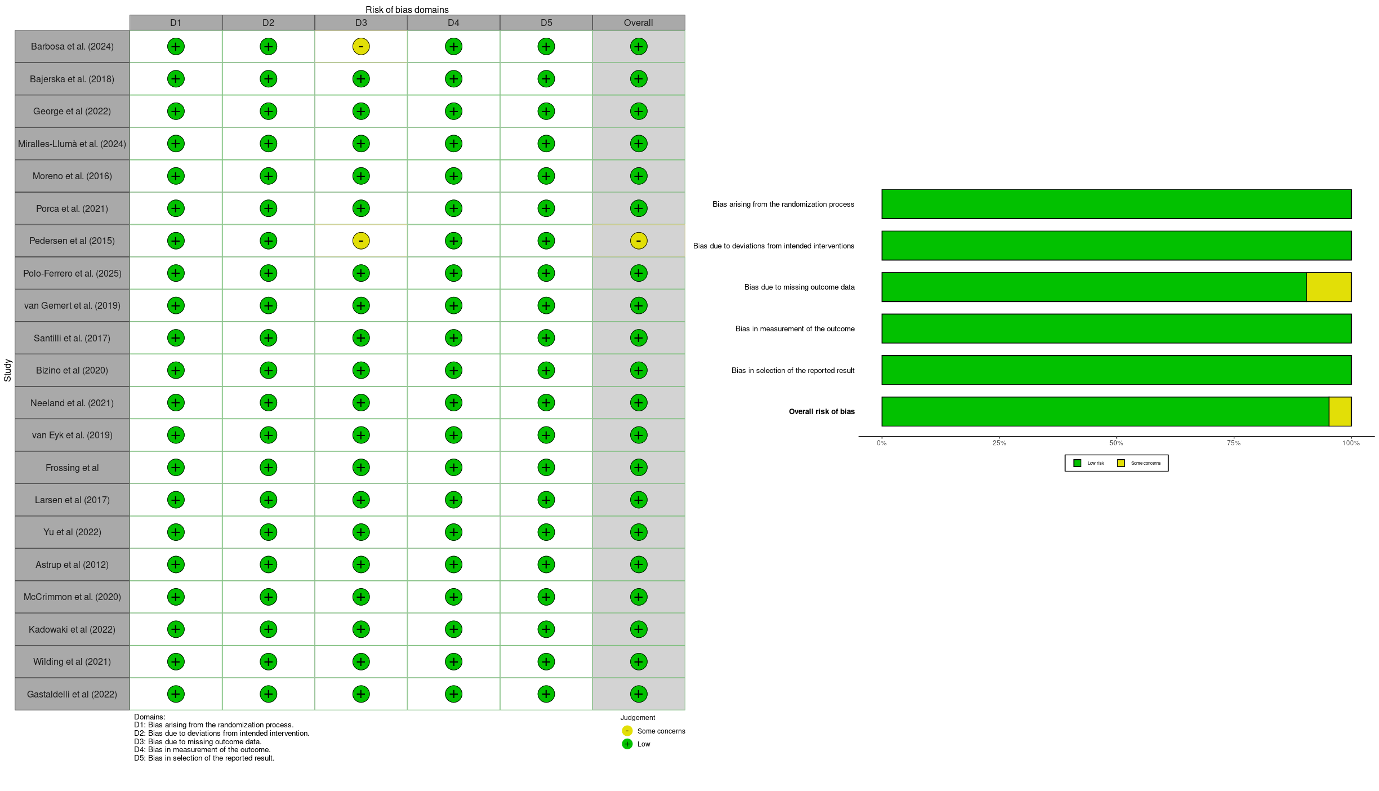

Supplement: Multimedia component 1 [file mmc1.docx]
